# Evaluation of leishmanicidal effect of *Euphorbia petiolata* extract by *in vivo* anti-leishmanial assay using promastigotes of *Leishmania major*

**Published:** 2018

**Authors:** Reza Kazemi Oskuee, Mahmoud Reza Jaafari, Mahdi Moghaddasi, Mahdi Rivandi, Fahime Afzaljavan, Mohammad Mohajeri, Mohammad Ramezani

**Affiliations:** 1 *Neurogenic Inflammation Research Center, Mashhad University of Medical Sciences, Mashhad, Iran*; 2 *Department of Medical Biotechnology, Faculty of Medicine, Mashhad University of Medical Sciences, Mashhad, Iran*; 3 *Nanotechnology Research Center, Pharmaceutical Technology Institute, Mashhad University of Medical Sciences, Mashhad, Iran*; 4 *School of Pharmacy, Mashhad University of Medical Sciences, Mashhad, Iran*; 5 *Department of Modern Sciences and Technologies, Faculty of Medicine, Mashhad University of Medical Sciences, Mashhad, Iran*; 6 *Pharmaceutical Research Centers, Pharmaceutical Technology Institute, Mashhad University of Medical Sciences, Mashhad, Iran*

**Keywords:** Leishmaniasis, Soxhlet extracts, Euphorbia petiolate, Promastigotes

## Abstract

**Objective:**

The extract of different species of *Euphorbia* has been successfully used as a remedy for treatment of cutaneous leishmaniasis. The aim of this study was to assess the *in vitro* leishmanicidal effect of *Euphorbia petiolata* (*E. petiolata*) extract.

**Materials and Methods:**

Ethanolic percolated and methanolic Soxhlet extract of *E. petiolata* on promastigotes of *L. major* at different concentrations of extracts, one positive control group and one negative control group as well as 1 solvent control were prepared and placed in 24-well plates that contained 40,000 parasites/well. Afterwards, plates were incubated at 25 ˚C for six days and number of parasites in each well were determined on days 2, 4 and 6 of the experiment.

**Results:**

Both percolated and Soxhlet extracts in methanol and DMSO solvents had significant effects (equal to that of amphotericin B) on promastigote form of parasite at the concentration of 1 mg/ml. At lower concentrations, the extracts of *E. petiolata* had favorable leishmanicidal activity and killed *L. major* promastigotes dose-dependently.

**Conclusion:**

Our results support the possibility of *E. petiolata* extracts application as an anti-leishmanial agent with similar effects to amphotericin B.

## Introduction

Various species of protozoa that belong to the genus *Leishmania *can cause leishmaniasis that is a chronic and non-contagious infection. The infected animals transfer disease to humans by the bite of a sand fly (Program for the surveillance and control of leishmaniasis 2002). More than 50% of new cases of leishmaniasis are cutaneous leishmaniasis (Jaafari et al., 2007 ▶; Jafari et al., 2005 ▶; Oskuee et al., 2014 ▶). It seems that cutaneous leishmaniasis (CL) is an important public health and public concern in many developing countries (Daryani et al., 2018 ▶; Hashiguchi et al., 2018 ▶). More than 90% of patients of CL are living in ten countries (i.e. Iran, Afghanistan, Iraq, Saudi Arabia, Algeria, Ethiopia, Sudan, Syria, Brazil, and Peru) (Armah et al., 2018 ▶; Douris et al., 2004 ▶; Paniz-Mondolfi et al., 2017 ▶; Singh et al., 2017 ▶). 

Anti-leishmanial agents are still very limited (Ansari et al., 2017 ▶). Pentavalent antimonial compounds have been used for treatment leishmaniasis treatment for more than 50 years (Sundar, 2001 ▶). N-methyl-glucamine antimoniate (Glucantime®) and sodium stibogluconate (Pentostam®) are available for treatment of leishmaniasis (Freitas-Junior et al., 2012 ▶). Available topical drugs have a very low leishmanicidal effect. Therefore, it is essential to develop new medications that are effective and safe for the treatment of leishmaniasis (Ansari et al., 2017 ▶; Berger et al., 2017 ▶; Camacho et al., 2003 ▶; Kassem et al., 2017 ▶). Natural products obtained from medicinal plants are regarded as new potential compounds for treatment of the disease (Jaafari et al., 2006 ▶; Jafari et al., 2005 ▶; Zahir et al., 2015 ▶). Plant extract or plant-derived compounds are likely valuable sources of new medicinal agents (Iranshahi et al., 2007 ▶). Some species of Euphorbia grow in Iran. *Euphorbia bungei *Boiss extract has been successfully used for the treatment of CL recently (Vasas and Hohmann, 2014 ▶). Previously, a dose-dependent antileishmanial activity of *E. myrsinites* extract was indicated (Jaafari et al., 2006 ▶; Jafari et al., 2005 ▶). Euphorbiaceae, the largest family of anthophyta, has 300 genera and 5000 species (Tewari et al., 2017 ▶; Yu et al., 2017 ▶; Zahidin et al., 2017 ▶). It was suggested that *Euphorbia *species have anti-inflammatory (Seebaluck-Sandoram et al., 2017 ▶; Wang et al., 2017 ▶), antiarthritic (Bani et al., 2007 ▶), antiamoebic (Ahmed et al., 2014 ▶), antispasmolytic (Jeyasankar and Elumalai, 2012 ▶), antiviral (Karimi et al., 2016 ▶; Mohammadi-Kamalabadi et al., 2014 ▶), hepatoprotective (Kaur et al., 2017 ▶; Seebaluck et al., 2015 ▶; Srirama et al., 2012 ▶), and antitumor activities (Alonso-Castro et al., 2012 ▶). The main classes of secondary metabolites present in *Euphorbia *species are alkaloids, terpenes, cyanogenic glycosides, glucosinolates, lipids and tannins (Bordoloi et al., 2017 ▶; Cordeiro Arruda et al., 2015 ▶; Galvão et al., 2017 ▶; Mohammadi-Kamalabadi et al., 2014 ▶; Ramalho et al., 2017 ▶; Xu et al., 2015 ▶). Studies have reported *Euphorbia petiolata* therapeutic effects such as antibacterial, tumor growth inhibition and free radical scavenging effects (Amirghofran et al., 2011 ▶; Kirbag et al., 2013 ▶; Nazemiyeh et al., 2010 ▶; Özbigin and Saltan Çitoǧlu, 2012 ▶). In this study, *in vitro* antileishmanial effects of the extracts of different aerial part of *E. petiolata* on promastigotes of *Leishmania major *were evaluated 

## Materials and Methods

**Plant Materials**

*E. petiolata* was collected in June–July 2015 from countryside of Mashhad (Khorasan Razavi Province, Iran). The aerial parts of the plant were dried in shade and then powdered. The plant material was identified by Herbarium of School of Pharmacy, Mashhad University of Medical Sciences, Mashhad, Iran and samples were conserved there with reference number 321.

**Preparation of extract**

*Soxhlet methanolic extract*: 

Fifty grams of the plant powder was extracted using 400 ml methanol for 24 hr by Soxhlet apparatus. The solvent was dried and removed under reduced pressure. The extract was kept refrigerated until use. 

*Macerated ethanolic extract:*

Fifty grams of the plant was extracted using 1000 ml ethanol (80% v/v) by maceration for three days. The extract was collected every 24 hr. The collected extracts were dried under reduced pressure and kept in refrigerator for further testing.

***Leishmania*** **parasites**

To obtain promastigotes of *L. major* strain MRHO/IR/75**/**ER, an infected BALB/c female mouse was selected and amastigotes were isolated from the lesions of the mouse. Then, amastigotes were transformed to promastigotes on Novy-MacNeal-Nicolle medium (NNN) and sub cultured in RPMI 1640 medium (containing 10% FCS, 2 mM glutamine, 100 U/ml of penicillin and 100 mg/ml of streptomycin) at 25 °C. The assay was performed according to a previously approved method (Iranshahi et al., 2007). Briefly, promastigotes in the stationary-phase were seeded at 40,000 parasite per well in a 24-well plate in RPMI-FCS. Methanolic or DMSO (as extract solvents) were added to each well to reach a final concentration of 1 mg/ml and serial two-fold dilutions thereof to reach final concentrations of 1, 0.5, 0.25, 0.125 and 0.0625 mg/ml. During a week of incubation at 25 C˚, the number of the parasites in each well was determined every two days under a microscope using Neubauer chamber (Hemocytometer). Amphotericin B (0.5mg/ml) was used as positive control. Culture media was used as negative control, and DMSO and methanol alone were used as solvent controls.

**Statistical analysis**

All experiments were performed in triplicate and data were expressed as mean±standard deviation (SD). Statistical analysis was performed using Prism Software Ver.5. Comparison between differences of means was made by one-way ANOVA analysis and a p-value≤0.05 was considered significant. The EC_50_ was determined by Litchfield and Wilcoxon method.

## Results

**Antileishmanial activity of macerated extract of** ***E. petiolata*** **in DMSO**

**Figure 1:**
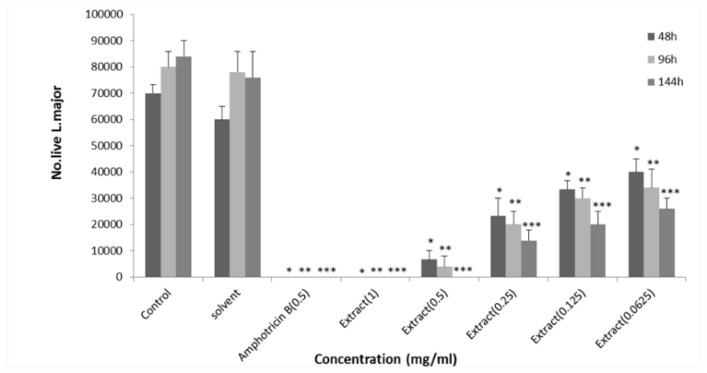
Effect of different concentrations of *E. petiolata* macerated extract in DMSO against *L. major* promastigotes after 2, 4 and 6 days of incubation. Each bar represents the mean+SD of the number of promastigotes in 24 wells. *p<0.05, **p<0.01, and ***p<0.0o1 as analyzed by Tukey-kramer test

*L. major* promastigotes were treated with amphotericin B (0.5 mg/ml) as well as macerated ethanolic extract of *E. petiolata* at the concentration of 1 mg/ml in DMSO. Lower doses of macerated ethanolic extract of *E. petiolata* in DMSO killed *L. major* promastigotes dose-dependently while DMSO did not have such effects on *L. majo*r promastigotes. The EC_50_ values for macerated ethanolic extract of *E. petiolata* in DMSO were 0.10, 0.058 and 0.034 mg/ml after 2, 4 and 6 days of incubation, respectively. The anti-leishmanial activity was lower after 6 days of incubation although it was not significantly different from that of days 2 and 4 (Figure 1).

**Antileishmanial activity of** **macerated extract** **of** ***E. petiolata*** **in methanol**

**Figure 2:**
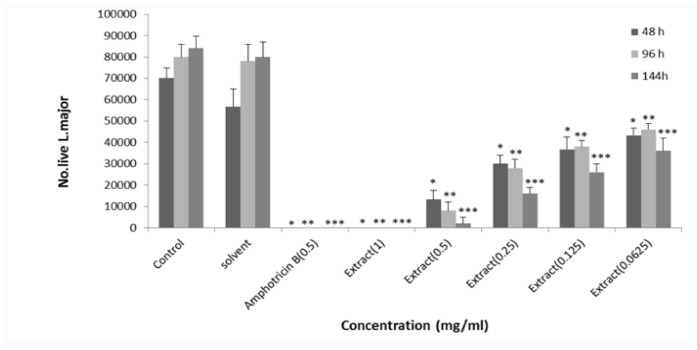
Effect of different concentrations of *E. petiolata *macerated extract in methanol against *L. major* promastigotes after 2, 4 and 6 days of incubation. Each bar represents the mean+SD of the number of promastigotes in 4 wells. *p<0.05, **p<0.01, and ***p<0.001 as analyzed by Tukey-kramer test

Amphotericin B (0.5 mg/ml) and macerated ethanolic extract of *E. petiolata* (1 mg/ml) in methanol killed all *L. major* promastigotes and lower doses of macerated ethanolic extract of *E. petiolata* in methanol killed *L. major* promastigotes dose-dependently while methanol did not have such effects on *L. major* promastigotes. The EC_50_ values for macerated ethanolic extract of *E. petiolata* in methanol were 0.123, 0.092 and 0.060 mg/ml after 2, 4 and 6 days of incubation, respectively. The anti-leishmanial activity was lower after 6 days of incubation although it was not significantly different from that of days 2 and 4 (Figure 2). 

**Antileishmanial activity of Soxhlet extract of** ***E*** ***petiolata* in DMSO**

**Figure 3:**
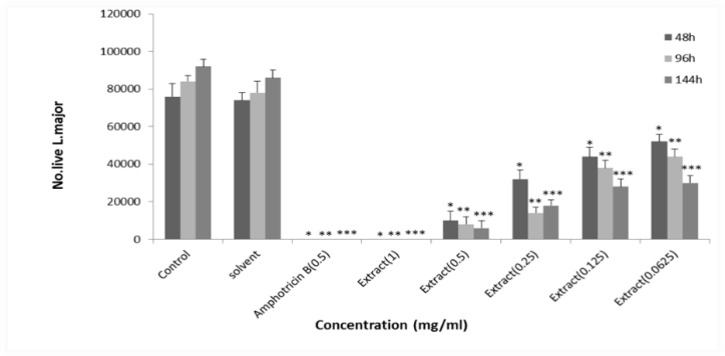
Effect of different concentrations of *E. petiolata* Soxhlet extract in DMSO against *L.* *major* promastigotes after 2, 4 and 6 days of incubation. Each bar represents the mean+SD of the number of promastigotes in 4 wells. *p<0.01, **p<0.01, and ***p<0.01 as analyzed by Tukey-kramer test

Different concentrations of Soxhlet methanolic extract of *E. petiolata* in DMSO killed *L. major* promastigotes dose-dependently. The EC_50_ values for Soxhlet methanolic extract of *E. petiolata* in DMSO were 0.118, 0.083 and 0.042 mg/ml after 2, 4 and 6 days of incubation, respectively (Figure 3).

**Anitileishmanial activity of Soxhlet extract of** ***E. petiolata*** **in** **methanol**

**Figure 4:**
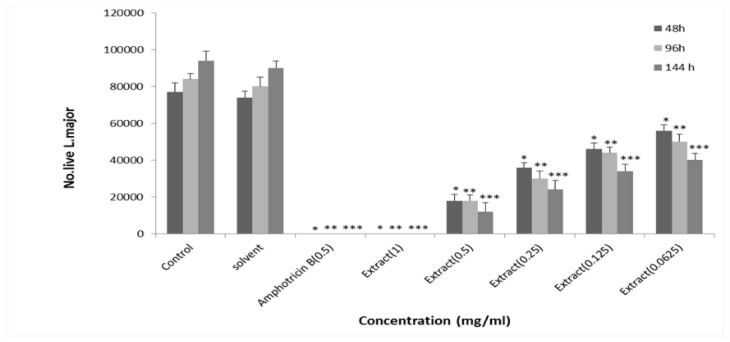
Effect of different concentrations of *E. petiolata* Soxhlet extract in methanol against *L. major* promastigotes after 2, 4 and 6 days of incubation. Each bar represents the mean+SD of the number of promastigotes in 4 wells. *p<0.05, **p<0.01, and ***p<0.001 as analyzed by Tukey-kramer test

Amphotericin B (0.25 mg/ml) and Soxhlet methanolic extract of *E. petiolata* (1 mg/ml) in methanol killed all *L. major* promastigotes and lower doses of macerated methanolic extract of *E. petiolata* in methanol killed *L. major* promastigotes dose-dependently. The EC_50_ values for Soxhlet methanolic extract of *Euphorbia petiolata* in methanol were 0.167, 0.118, 0.066 mg/ml after 2, 4 and 6 days of incubation, respectively (Figure 4).

## Discussion

Despite tremendous progress in diagnosis and treatment of human diseases, the plant(s)/plant-derived compounds are commonly used to treat some disorders such as cutaneous leishmania without any experimental evidence to explain their activity. Cutaneous leishmaniasis has become a main challenge in many developing countries. Given that chemotherapy is painful and sometimes not effective, patients tend to use herbal remedies. One of those claims is indicating the extract and latex of *E. petiolata* as a topical treatment against cutaneous leishmanias. To asses that, in this study *E. petiolata* extracts were utilized against promastigotes to analyze their efficacy *in vitro*. Both macerated and Soxhlet extracts of the aerial parts of *E. petiolata* were prepared and tested against promastigotes of *L. major*. All tested concentrations of both extracts exhibited leishmanicidal activity after 2, 4 and 6 days of incubation. Although the number of live promastigotes after 6 days of incubation was lower than those of incubation days 2 and 4, but no significant differences among these days were observed. It could be suggested that the active constituents of the extracts were not thermally labile. Both macerated and Soxhlet extracts at highest concentration (1 mg/ml) were able to kill all of promastigotes like amphotericin whereas in similar studies on *E. myrsinites *and *E. microciadia*, mortality ranged between 75 and 100% at different time points (Jafari et al., 2005 ▶). The level of mortality at second dilution of macerated extract in DMSO was higher than those of *E. microciadia* (65.9-94.2) and *E. myrsinites *(61-92). Statistically, there was no difference between macerated and Soxhlet extracts against promastigotes but comparison of diagrams pointed out that macerated extract was more effective than Soxhlet extract in killing promastigotes. This result may be due to utilization of heat in preparation of the Soxhlet extract that can reduce the effectiveness of materials. The EC_50_ values of both macerated and Soxhlet extracts were in the range of 34-167 µg/ml which could be considered a moderate anti–leishmanial activity value. Despite the fact that natural compound with EC_50_ higher than 25 µM is considered inactive and concentrations obtained in this study were not typically low, the active leishmanicidal components may be present at very low concentration warranting the need for purification of active constituents of *E. petiolata *(Jaafari et al., 2007 ▶). Phytochemical screening had shown the presence of alkaloids, saponins, tannins and flavonoids, where flavonoids and tannins were the major constituents. Several reports indicated alkaloids (Ferreira et al., 2010 ▶), chalcones (Flores et al., 2007 ▶), triterpenoids (Kennedy et al., 2011 ▶), saponins (Mskhiladze et al., 2008 ▶) and polyphenols (Hay et al., 2008 ▶) as the leishmanicidal constituents of some plants. According to primitive phytochemical screening, the antileishmanial effects could be due to the presence of flavonoids, alkaloids and saponins. However, fractionation of *E. petiolata* is essential to discover the component(s) with leishmanicidal activity.

Our results support the possibility of application of *E. petiolata* extract as an anti-leishmanial agent with similar effects to amphotericin B. Above all, percolated extract was more effective than Soxhlet extract. Moreover, effects of extracts in DMSO are more marked than those of the extracts in methanol. Further *in vivo* experiments in animal models are recommended before performing clinical evaluation of *E. petiolata *effects.
